# Identification of HLA class II-restricted T cell receptors against shared PIK3CA mutations in patients with epithelial cancers

**DOI:** 10.1007/s00262-025-04156-3

**Published:** 2025-12-18

**Authors:** S. M. Rafiqul Islam, Samantha Seitter, Maria Parkhurst, Frank J. Lowery, Miaki Fukuhara, Sanghyun P. Kim, Noam Levin, Jared J. Gartner, Satyajit Ray, Todd Prickett, Victoria Hill, Paul F. Robbins, Stephanie L. Goff, Steven A. Rosenberg, Nikolaos Zacharakis

**Affiliations:** National Cancer Institute, Surgery Branch, National Institutes of Health, Bethesda, MD USA

**Keywords:** T cell, TCR, PIK3CA, immunotherapy

## Abstract

*PIK3CA* is the third most common mutated gene in epithelial cancers, behind only *TP53* and *KRAS*. Somatic *PIK3CA* mutations occur predominantly in the protein’s hotspot locations H1047, E545, E542 and N345, and evidence of those alterations eliciting immune responses has been limited. HLA class I-restricted TCRs against E545K and H1047L have, only recently, been identified following in vitro sensitization (IVS) with peripheral blood lymphocytes (PBL) from normal donors. In this study, we examined the immunogenicity of PIK3CA in patients with epithelial cancers of diverse histology, by testing TIL and antigen-experienced PBL against autologous hotspot PIK3CA mutations. Two distinct TCRs specific to PIK3CA^N345K^, restricted by HLA-class II pair DPB1*04:01/DPA1*01:03, were identified in a patient with colon cancer, following independently a TIL screen and an IVS of memory CD4 + *T* cells from the peripheral blood. Patient PBL engineered to express each TCR suppressed the in vitro growth of two cell lines modified to express the DPB1*04:01/DPA1*01:03 pair and full length PIK3CA^N345K^ protein. A TCR specific to the PIK3CA^E545K^, restricted by HLA-DRB1*04:01, was also identified following IVS of memory CD4 + PBL from another patient, with rectal cancer. Autologous PBL, gene engineered to express the PIK3CA^E545K^-specific TCR, suppressed the in vitro growth of two cancer cell lines, which endogenously expressed the PIK3CA^E545K^ neoantigen. This study identified three PIK3CA-specific TCRs against two shared mutations, restricted by common HLA-class II molecules, using antigen-experienced TIL and memory PBL from patients with epithelial cancers and may further allow the development of off-the-shelf *T* cell immunotherapy strategies targeting PIK3CA.

**Supplementary Information:**

The online version contains supplementary material available at 10.1007/s00262-025-04156-3.

## Introduction

Adoptive *T* cell therapy using tumor-infiltrating lymphocytes (TILs) and *T* cell receptor (TCR) engineered *T* cells can mediate durable tumor regressions in patients with metastatic epithelial cancers [1–7]. The targets for these treatments are either spontaneous, often private, or shared hotspot somatic mutations. Common oncogenic gene mutations, such as those in *TP53*, *KRAS*, *PIK3CA* and *EGFR* can give rise to shared neoantigens that are usually found to be clonal as they confer a tumorigenic phenotype on the cancer cells.

*PIK3CA* is the third most mutated gene in epithelial cancers, after only *TP53* and *KRAS* [8]. In breast cancer, approximately 32–37% of all patients with advanced disease harbor a *PIK3CA* mutation, with the most frequent alterations being p.H1047R (23.1%), p.E545K (23.1%), p.E542K (16.3%), p.H1047L (3.2%) and p.N345K (3.1%, among all *PIK3CA* mutations) [8, 9].

*T* cell receptors (TCR) that recognize shared neoantigens can be inserted into autologous peripheral blood lymphocytes (PBL) and can mediate an in vivo anti-tumor effect [2, 4, 7, 10]. Recent studies reported that hotspot *PIK3CA* mutations can be immunogenic and could represent an attractive target for *T* cell-based cancer immunotherapies [11–14]. The identification of PIK3CA-specific TCRs in these studies involved in vitro sensitization of bulk normal donor PBL or mouse vaccination models. All TCRs reported to date, targeting mutant PIK3CA are HLA class I-restricted, and it is not known whether *PIK3CA* mutations can give rise to reactive CD4 + *T* cells. In the current study, we investigated the presence of antigen-experienced *T* cells in TIL and memory PBL from patients with cancer, against shared *PIK3CA* mutations. We report the identification of two TCRs recognizing the PIK3CA^N345K^ neoantigen and one TCR recognizing the PIK3CA^E545K^ neoantigen, both of which were restricted by common HLA-class II alleles.

## Results

### *Identification of PIK3CA*^*N345K*^*-specific TCRs in TIL and peripheral blood from a patient with colon cancer*

**Fig. 1:**
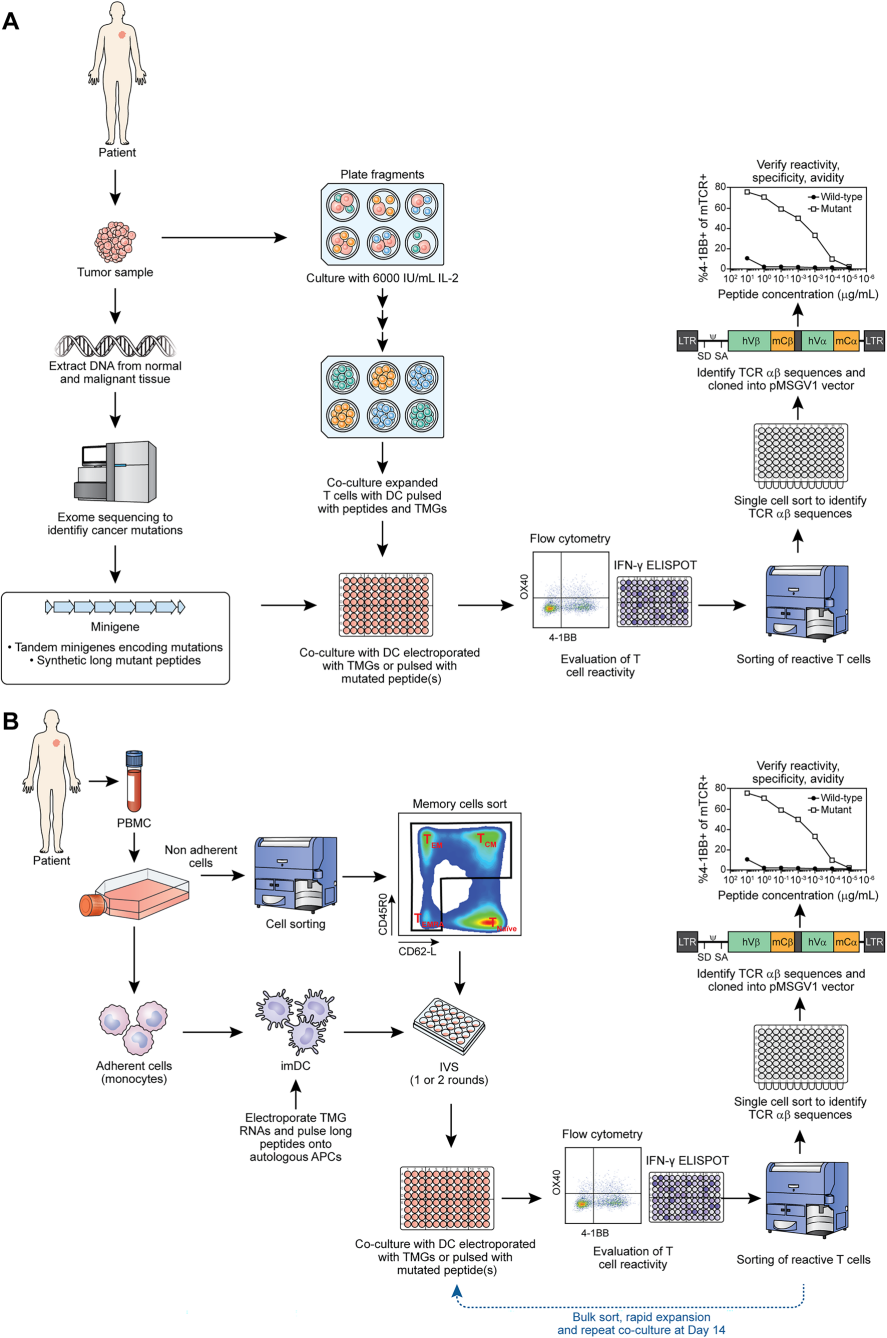
Schemas for the identification of PIK3CA-reactive *T* cells in TIL and PBL from patients with cancer and treatment with PIK3CA-TCR- transduced PBL. **A** Identification of PIK3CA-specific *T* cells via pipeline TIL screen against autologous somatic mutations. Following identification of reactive *T* cells and isolation/verification of the alpha/beta-TCR chains, the PIK3CA-specific TCR was retrovirally transduced into autologous PBL for functional characterization. **B** Identification of PIK3CA-specific *T* cells from memory PBL after in vitro stimulation with peptides and TMG encoding shared PIK3CA mutations. The PIK3CA-reactive cells were sorted by flow cytometry and alpha/beta TCR chains were isolated and tested for specificity

TIL from a patient with colon cancer (Pt 4367) were screened against all autologous somatic mutations, including the PIK3CA mutant p.N345K, as part of our main pipeline TIL screen, which has been previously described [1, 3, 15]. Briefly, a metastatic lesion was surgically excised, and TIL were isolated by placing 24 tumor fragments into culture in presence of high concentration of IL-2. Simultaneously, the tumor was analyzed for identification of autologous exonic somatic mutations, which were synthesized in the form of long peptides or tandem mini genes (TMG) with the mutation in the middle of a 25mer peptide or mini gene surrounded by the normal gene sequence. Finally, TIL were co-cultured with autologous antigen presenting cells (APCs) which were pulsed with autologous long peptides or electroporated with TMGs, and *T* cell activation is measured by upregulation of 4-1BB (CD137) and IFN-γ secretion (Fig. 1A).

**Fig. 2:**
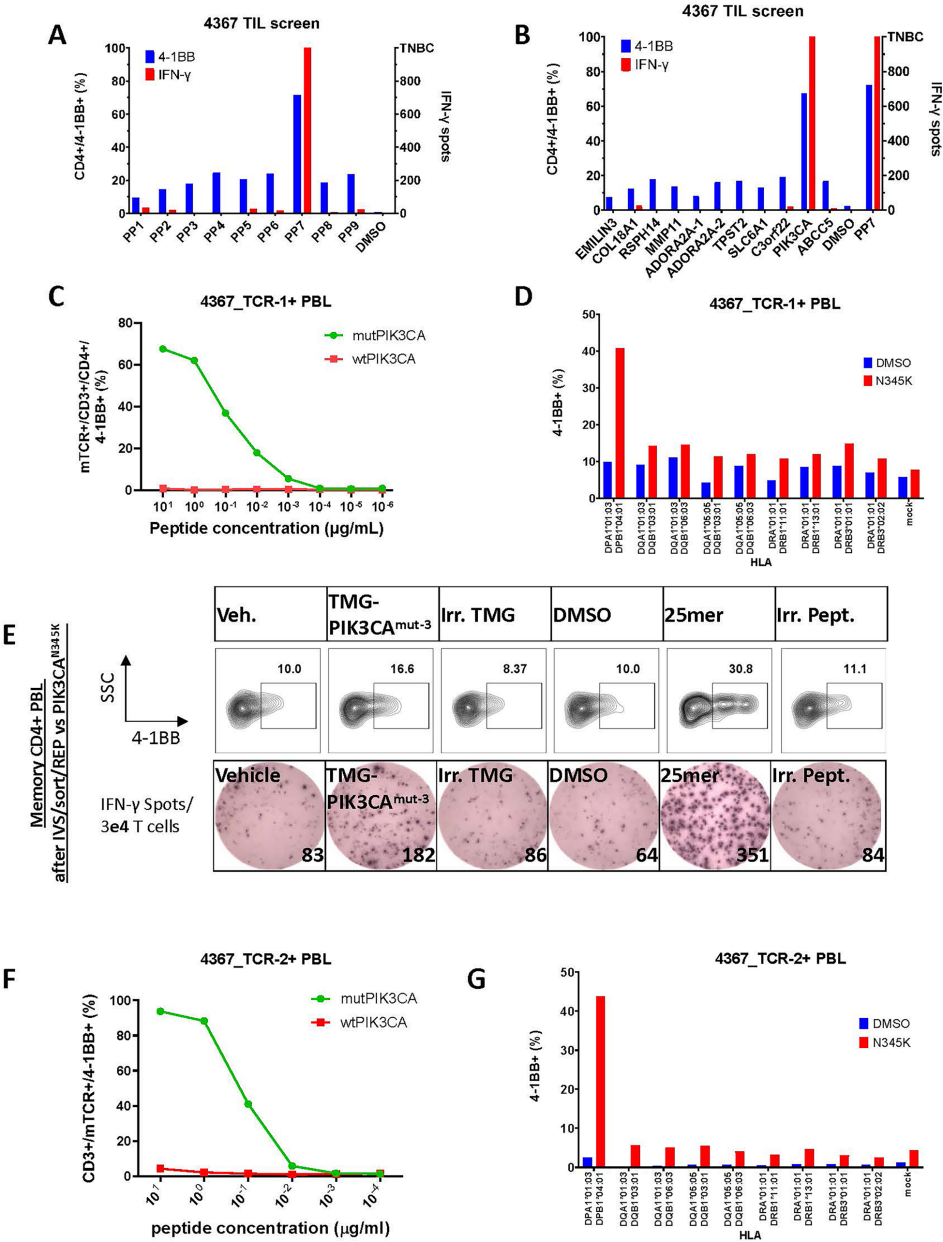

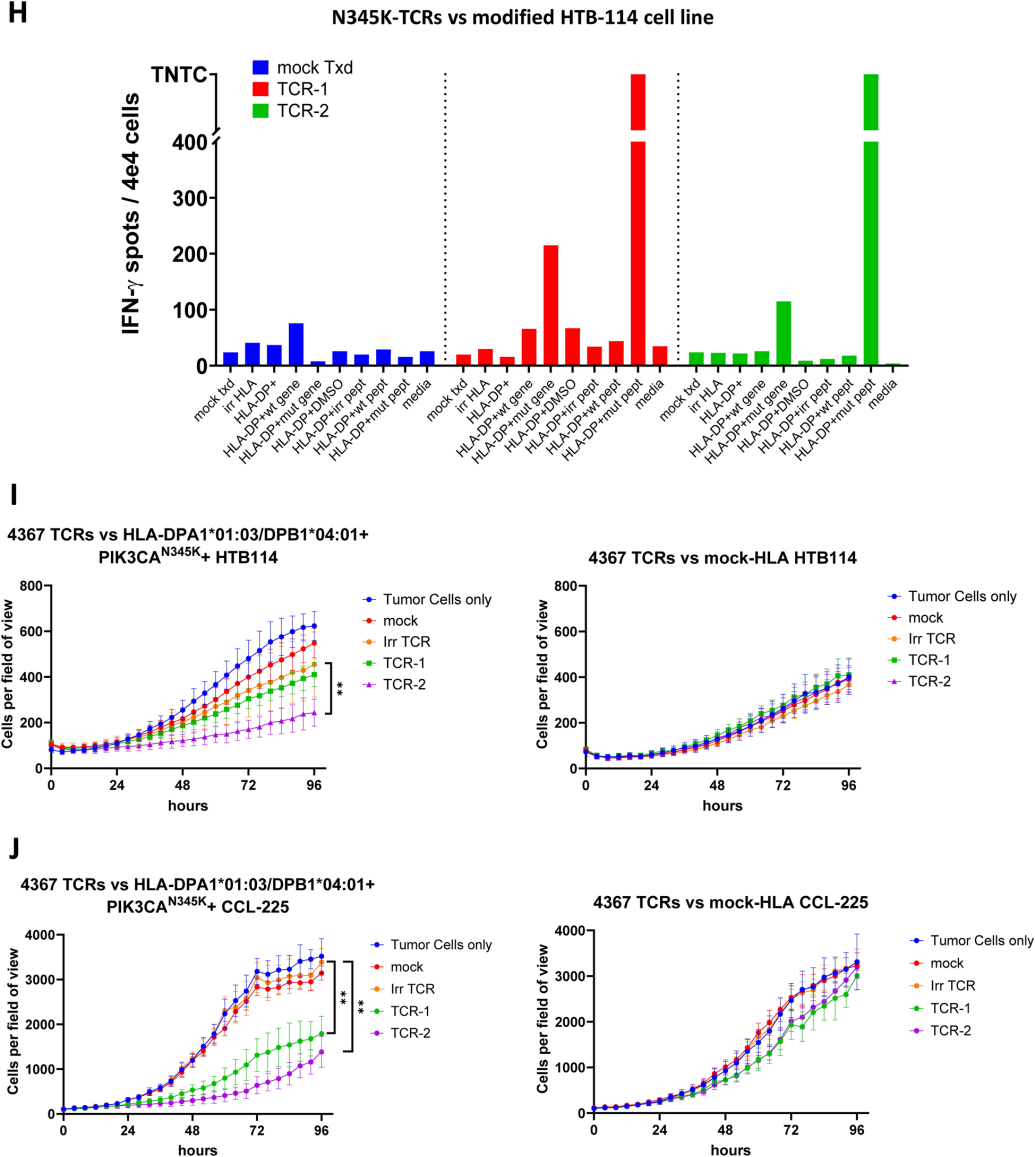
Identification of PIK3CA-N345K-specific TCRs in TIL and peripheral blood from a patient with colon cancer. **A** 4-1BB (CD137) upregulation (of CD4 + *T* cells) and IFN- *γ* secretion (ELISPOT assay) by 4367 TIL fragment F10, following coculture with autologous DCs pulsed with pools (PP) of long (≤ 25AA) mutant peptides. **B** 4-1BB (CD137) upregulation (of CD4 + *T* cells) and IFN- *γ* secretion (ELISPOT assay) by 4367 TIL fragment F10, following coculture with autologous DCs pulsed with the individual peptides comprising the peptide pool PP7. **C** 4-1BB (CD137) upregulation of mTCR + /CD3 + /CD4 + PBL retrovirally transduced with 4367_TCR-1 in response to titrated concentration of mutant or wild type long PIK3CA^N345K^ peptide pulsed on autologous DCs. This experiment was performed once. **D** 4-1BB (CD137) upregulation of CD3 + /mTCR + PBL from healthy donor-1, retrovirally transduced with 4367_TCR-1 in response to COS7 cells transfected with each of the 4367 class II HLA molecules and pulsed with the PIK3CA^N345K^ peptide or vehicle (DMSO). **E** 4-1BB (CD137) upregulation (of CD4 + *T* cells) and IFN- *γ* secretion (ELISPOT assay) by 4367 memory CD4 + PBL following IVS with PIK3CA^N345K^ 25mer, sort of the reactive *T* cells against the PIK3CA^N345K^ 25mer, rapid expansion and overnight co-culture with DCs pulsed (peptides or DMSO) or transfected (TMGs or vehicle) with the indicated conditions. **F** 4-1BB (CD137) upregulation of CD3 + /mTCR + PBL from healthy donor-1, retrovirally transduced with 4367_TCR-2 in response to titrated concentration of mutant or wild type long PIK3CA^N345K^ peptide pulsed on autologous DCs. **G** 4-1BB (CD137) upregulation of CD3 + PBL retrovirally transduced with 4367_TCR-2 in response to COS7 cells transfected with each of the 4367 class II HLA molecules and pulsed with the PIK3CA^N345K^ peptide or vehicle (DMSO). This experiment was performed once. **H** IFN-*γ* secretion (ELISPOT assay) of PBL retrovirally transduced with either 4367_TCR-1 or _TCR-2 following overnight co-culture with modified (as indicated) HTB-114 cell line. E:T ratio = 1:3. TNTC: Too numerous to count. This experiment was performed once. In vitro tumor cell killing assays with autologous PBL retrovirally transduced with either 4367_TCR-1, 4367_TCR-2 or irrelevant TCR (Irr TCR) against modified **I** HTB-114 and **J** CCL 225 cell lines. The target cells were either transduced with HLA- DPA1*01:03/DPB1*04:01 and the full length mutated PIK3CA gene (left panel) or mock-transduced (right panel). Effector to target ratio: 10:1. Mean with standard deviation of six culture replicates is graphed. **P < 0.01 as calculated by Mann–Whitney test comparing slopes between TCR-1 or TCR-2 and Irr TCR. Only statistically significant differences are shown

In the TIL screen for patient 4367, one TIL subculture (F10) showed *T* cell activation following co-culture with APCs pulsed with peptide pool 7 (PP7) (Fig. 2A). When individual peptides consisting the PP7 were tested, F10 TIL showed upregulation of 4-1BB and secretion of IFN-γ against the mutant PIK3CA^N345K^ peptide (ALRIKILCATYVKVNIRDIDKIYVR) (Fig. 2B). Following fluorescence-activated cell sorting (FACS) of the reactive *T* cells and TCR-specific sequencing, the isolated TCR (4367_TCR-1) (Suppl. Table S1) was resynthesized, introduced into donor *T* cells and was tested against titrated concentration of the mutant and wild type PIK3CA peptide, exhibiting specificity to PIK3CA^N345K^ mutant peptide in as low as 1 ng/ml (EC50 = 80 ng/ml) (Fig. 2C). The HLA-restriction for the anti-PIK3CA 4367_TCR-1 was determined to be the HLA-class II pair DPA1*01:03/DPB1*04:01, following co-culture with PIK3CA^N345K^ pulsed COS7 cells transfected with each of the patient’s HLA-class II molecules (Fig. 2D and Suppl. Fig. S1A).

To evaluate the presence of PIK3CA-specific *T* cells in the peripheral blood, memory T lymphocytes were isolated from PBL by sorting the CD62L + CD45RO + (Tcm), CD62L-CD45RO + (Tem) and CD62L-CD45RO- (Temra) *T* cells and then subjected them to in vitro stimulation (IVS) with mutant PIK3CA long peptides or TMGs or predicted minimal epitopes, an approach that has been previously described [16, 17] and illustrated in Fig. 1B.

In particular, memory CD4 + and CD8 + *T* cells from the peripheral blood of patient 4367 were subjected to one round of IVS and the resulting populations were screened for reactivity against PIK3CA^N345K^-expressing TMG and peptide. Stimulated CD4 + *T* cells were found to be reactive in low frequency against the PIK3CA^N345K^ mutated TMG (CD4 + /4-1BB + : 1.4% TMG(PIK3CA) vs 0.8% vehicle) (Suppl. Fig. S1B). The reactive *T* cells with the highest 4-1BB + expression were sorted (*n* = 59) and were subjected to a REP to increase the cell number. The expanded *T* cell population was verified to recognize the p.N345K 25AA-long peptide as shown by flow cytometry and IFN-γ secretion (Fig. 2E). TCR sequencing of the sorted reactive *T* cells resulted in the reactive TCR sequence (4367_TCR-2) which was unique, with alpha and beta-TCR chains distinct from the TIL-derived TCR (4367_TCR-1) (Suppl. Table S1). The 4367_TCR-2 was resynthesized, introduced into donor *T* cells and recognized the PIK3CA^N345K^ mutation, and not the wild type peptide, in concentrations as low as 10 ng/ml (EC50 = 126 ng/ml) (Fig. 2F and Suppl. Fig. S1C). The HLA-restriction was determined by PIK3CA^N345K^-pulsed single-HLA transduced COS7 and was the same as for 4367_TCR-1, the HLA-class II pair DPA1*01:03/DPB1*04:01 (Fig. 2G).2

Next, we evaluated the recognition of tumor cells that can express and process the mutant PIK3CA protein by the two specific 4367 PIK3CA^N345K^-TCRs. No autologous or allogeneic tumor cells with the mutant PIK3CA^N345K^ and DPA1*01:03/DPB1*04:01 were available. Therefore, we engineered the human uterus tumor cell line, HTB-114 to express the HLA pair DPA1*01:03/DPB1*04:01, with or without co-expression of the mutant full length N345K PIK3CA protein (Suppl. Fig. S2). Healthy donor PBL transduced with either TCR-1 or TCR-2 recognized the modified tumor cell line only when the appropriate restriction element was present and the mutant protein or peptide was endogenously expressed or pulsed, respectively (Fig. 2H). In a tumor killing assay, however, only the 4367_TCR-2-expressed *T* cells significantly (*p* = 0.004) suppressed the growth of the HTB-114 tumor cells, gene-engineered to co-express the DPA1*01:03/DPB1*04:01 and the mutant PIK3CA^N345K^ protein (Fig. 2I). We created a second model by engineering the colon adenocarcinoma cell line CCL-225 to overexpress the DPA1*01:03/DPB1*04:01 and the mutant full length PIK3CA^N345K^ protein. In a tumor killing cell assay, both the 4367_TCR-1 and TCR-2 significantly (*p* = 0.004) suppressed the growth of the tumor cells in vitro (Fig. 2I).

In summary, our findings suggest that both 4367_TCR-1 and TCR-2 specifically recognized the PIK3CA^N345K^ mutation, shared the same HLA-class II restriction (HLA-DPA1*01:03/HLA-DPB1*04:01), and recognized and suppressed gene-engineered tumor cell lines that were capable of immunologically processing and presenting the mutated PIK3CA antigen.

### *Identification of PIK3CA*^*E545K*^*-specific TCR in the peripheral blood from a patient with rectal cancer*

**Fig. 3:**
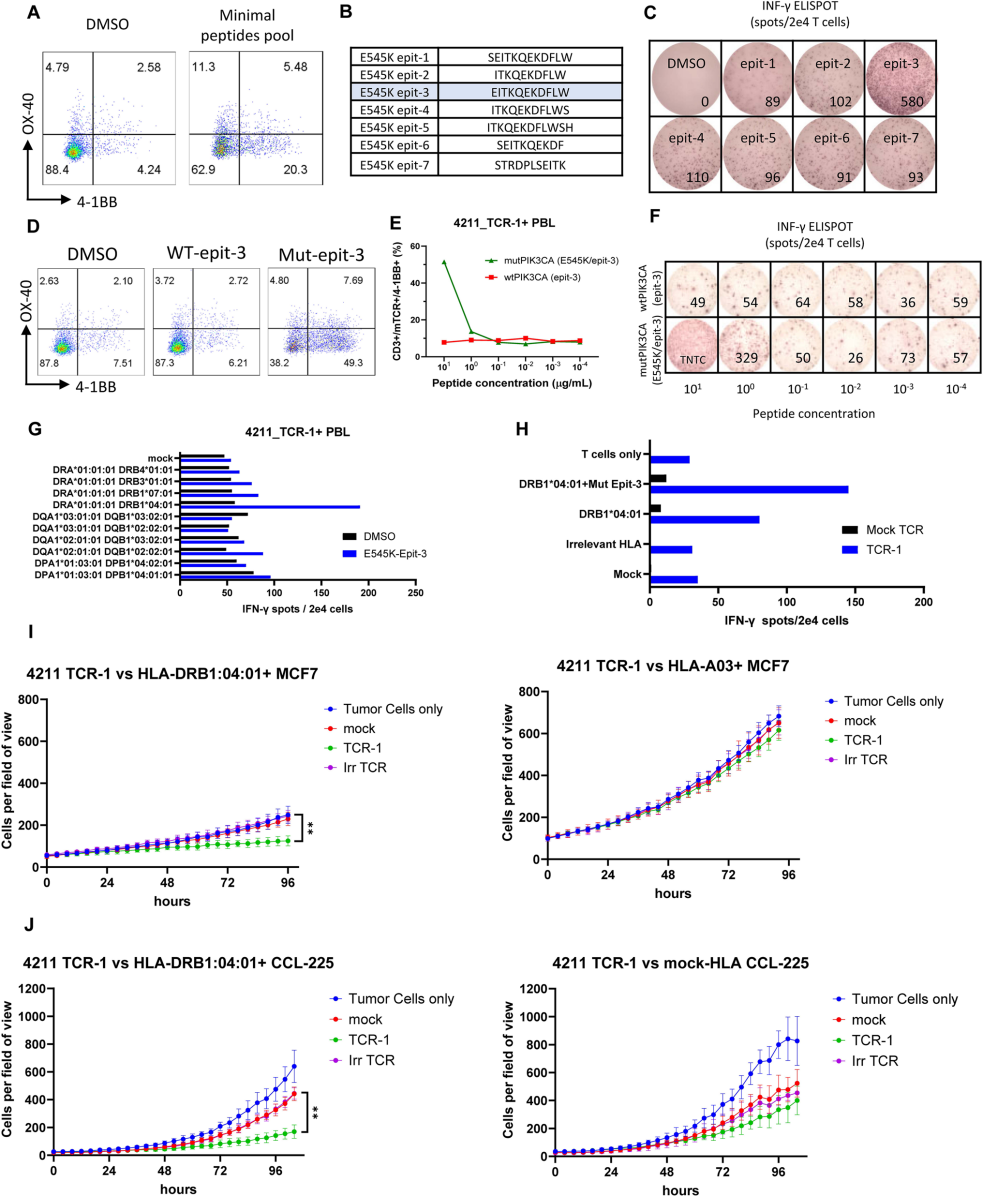
Identification of PIK3CA-E545K-specific TCR in the peripheral blood from a patient with rectal cancer.** A** 4-1BB (CD137) and OX-40 (CD134) upregulation by 4211 memory CD4 + PBL following two rounds of IVS with PIK3CA^E545K^ predicted minimal peptide pool and overnight co-culture with autologous DCs pulsed with vehicle (DMSO) or the predicted minimal peptide pool.** B** Table with the amino-acid sequences of short peptides (10-12AA) included in the examined predicted minimal peptide pool. **C** IFN-*γ* secretion (ELISPOT assay) after overnight co-culture of the CD4 + PBL described in A with autologous DCs pulsed with the individual peptides of the predicted minimal peptide pool as listed in B. **D** 4-1BB (CD137) and OX-40 (CD134) upregulation by 4211 memory CD4 + PBL after overnight co-culture with autologous DCs pulsed with vehicle (DMSO), wt-E545K_epit-3 (EITEQEKDFLW) or mut-E545K_epit-3 (EITKQEKDFLW). **E**, **F** 4-1BB (CD137) upregulation (of CD3 + /mTCR +) and IFN- γ secretion (ELISPOT assay) of PBL from healthy donor-1, retrovirally transduced with 4211_TCR-1, in response to titrated concentration of mutant or wild type PIK3CA^E545K_epit−3^ peptide pulsed on autologous DCs. TNTC: Too numerous to count. **G** The IFN- secretion (ELISPOT assay) of PBL retrovirally transduced with 4211_TCR-1 in response to COS7 cells transfected with each of the 4211 class II HLA molecules and pulsed with the PIK3CA^E545K_epit−3^ peptide or vehicle (DMSO). This experiment was performed once.** H** IFN-*γ* secretion (ELISPOT assay) of PBL from healthy donor-1 either mock-transduced or with 4211_TCR-1 following overnight co-culture with modified (as indicated) MCF-7 cell line. In vitro tumor cell killing assays with autologous PBL retrovirally transduced with 4211_TCR-1 or irrelevant TCR (Irr TCR) against **I** MCF-7 and **J** CCL225 cell lines. The target cells were either transduced with HLA- DRB1*04:01 (left panel) or with irrelevant HLA-A*03 (top right) or mock-transduced (bottom right). Effector to target ratio: 10:1. Mean with standard deviation of six culture replicates is graphed. **P < 0.01 as calculated by Mann–Whitney test comparing slopes between TCR-1 and Irr TCR. Only statistically significant differences are shown

Identification of *T* cells targeting the common PIK3CA mutation E545K was attempted in TIL and the peripheral blood from a patient with metastatic rectal cancer (Pt 4211). No reactive *T* cells were detected in the TIL when screened against all of the autologous neoantigen candidates, including a minigene encoding the candidate PIK3CA neoantigen and the corresponding 25mer peptide. Next, memory CD4 + and CD8 + *T* cells from the same patient were sorted by FACS based on the CD62L and CD45RO surface expression as described above for patient 4367. Sorted *T* cells were subjected to two subsequent rounds of IVS with long peptides, predicted minimal epitopes and a TMG encoding the PIK3CA^E545K^ mutation. At the end of the IVS, memory CD4 + *T* cells, which were stimulated twice with a peptide pool consisting of minimal epitope peptides, showed upregulation of the activation surface markers OX-40 and 4-1BB following overnight co-culture with autologous APCs pulsed with the respective peptide pool (Fig. 3A). The co-culture was repeated with individual minimal peptides from the reactive pool, revealing that the CD4 + *T* cells recognized only the E545K_epit-3 (EITKQEKDFLW) (Fig. 3B-C). This recognition was specific to the mutant and not the wild-type peptide (Fig. 3D). This was further validated following isolation of the PIK3CA^E545K^-reactive TCR sequence (4211_TCR-1) (Suppl. Table S1), resynthesis and introduction into healthy donor PBL and testing against titrated concentrations of the mutant and wild type short peptides, in which only the mutant peptide was recognized (EC50 = 2.4 μg/ml) (Fig. 3E-F and Suppl. Fig. S3A). Finally, we determined by single HLA-transduced COS7 cells that this PIK3CA^E545K^ reactivity was restricted by the HLA-class II molecule DRB1*04:01 (Fig. 3G).

To evaluate the recognition of tumor cells that endogenously expressed the mutant PIK3CA protein, 4211_TCR-1 was tested against the breast cancer derived cell line MCF-7 which naturally expresses the PIK3CA^E545K^ protein but lacks the DRB1*04:01 allele. 4211-TCR-1-transduced PBL were activated by co-culture with the MCF-7 only when the DRB1*04:01 was introduced in the tumor cell line, while irrelevant HLA introduction resulted only in baseline secretion of IFN-γ by the TCR-engineered *T* cells (Fig. 3H and Suppl. Fig. S3B).

This recognition of the engineered MCF-7 was further investigated in tumor cell killing assays where autologous PBL transduced with the 4211_TCR-1 suppressed (*p* = 0.0065) the growth of the MCF-7 cell line only when the appropriate HLA-DRB1*04:01 but not when the control HLA-A*03 was introduced (Fig. 3I, Suppl. Fig. S4). Similarly, in a second tumor model, only *T* cells transduced with the PIK3CA^E545K^-specific TCR, suppressed (*p* = 0.004) the growth of the cell line CCL-225, which also naturally expressed the E545K mutant protein, following transduction with HLA-DRB1*04:01 (Fig. 3I).

Thus, an HLA-class II restricted TCR, specific to the PIK3CA^E545K^ mutation was identified and isolated from memory PBL from a patient with rectal cancer and was able to recognize two cell lines endogenously expressing the mutant PIK3CA^E545K^ protein, following introduction of the HLA restriction element DRB1*04:01.

The presence of PIK3CA-specific TCR in TIL and memory PBL was investigated in a total of 30 patients with epithelial cancers of diverse histology, which expressed any of the five most frequent hotspot *PIK3CA* mutations (Table S3). Autologous TIL were screened against either the autologous candidate neoantigens, including PIK3CA or with dedicated screens against specific PIK3CA TMGs, long peptides and predicted short peptides. For 16 of these patients, TIL were also subjected to IVS with the autologous PIK3CA reagents – in addition to the memory PBL IVS such as these described above. In summary, at least 2 different investigations for *T* cell reactivity against a PIK3CA mutation applied to all 30 patients and both TIL and PBL IVS were performed in 16 of those patients. While several methodologies applied against several hotspot *PIK3CA* mutations, no additional TCRs against p.N345K, p.E545K or other hotspot PIK3CA neoepitopes were identified, indicating that reactivity against these candidate neoepitopes was not prevalent in this group of patients with metastatic cancer (Table S3).

## Discussion

The development of TIL-based immunotherapies against highly personalized neoantigens can mediate durable responses in solid cancers, predominantly in melanoma and recently shown in patients with epithelial cancers [1, 3, 5, 18]. However, the process remains lengthy (usually months) and laborious, often surpassed by rapid progression of disease in patients in an already advanced stage of cancer. The need for off-the-shelf cell-based immunotherapies requires the presence of a frequently occurring tumor target. Gene-engineered autologous PBL with an allogeneic TCR against the most common mutated genes, *TP53* and *KRAS* has resulted in encouraging clinical responses, although the responses observed to date have been short-lived [2, 4, 7]. While it may be possible to enhance the function of these *T* cells by developing methods to increase their anti-tumor functions or maintaining a younger phenotype, the identification of other commonly shared tumor targets will provide the breadth and flexibility of treatments to a larger number of patients.

This report provides evidence that three HLA-class II restricted TCRs can recognize the shared PIK3CA neoantigens, p.E545K and p.N345K. Recently, TCRs against PIK3CA^E545K^ or PIK3CA^H1047L^ have been reported, generated following IVS of healthy donor PBL or by vaccination of mice [11–14]. Our approach was focused on patients who already carried the mutant PIK3CA protein and all three TCRs were identified and isolated from antigen-experienced, memory *T* cells either in the PBL or TIL from these patients. This study reports two unique TCRs against the PIK3CA^N345K^ gene product, encoded by the 5th most common *PIK3CA* mutation, comprising 3% of all *PIK3CA* missense mutations, and is expressed in approximately 0.38% of all patients with advanced cancer [8]. Both TCRs are restricted by the highly frequent class II HLA-DPB1*04:01 which is expressed by about 85% of all US Caucasian population (Table S4) [9, 19]. Therefore, these two TCRs against PIK3CA^N345K^ are estimated to potentially be applicable in a target population of 0.32% of Caucasian patients with advanced cancer in the US (Table S4).

In addition, this study describes one TCR against the PIK3CA^E545K^ mutant gene product, one of the most frequent *PIK3CA* mutations that represents 23% of all *PIK3CA* mutations and is expressed by 2.8% of all patients with advanced cancer [8]. This TCR can potentially be applicable in an estimated target population of 0.48–0.56% of the US Caucasians, considering the frequency of the PIK3CA^E545K^ mutation and the estimated 17.3–20% of the US Caucasian population who expresses the appropriate HLA-restriction molecule, HLA-DRB1*04:01 (Table S4) [19].

All three TCRs described by this study were derived from CD4 + *T* cells and this was likely to be the major factor for the limited avidity and cytotoxicity against tumor cell lines in vitro. Notably, the 4211_TCR-1, when expressed on donor PBL, showed very low avidity against the p.E545K epitope as measured by titrated peptide concentrations. However, these cells suppressed the growth of two tumor cells line which endogenously expressed the mutant protein, demonstrating their potency to recognize tumor albeit with limited cytotoxic efficacy. Similar tumor cell killing results were observed with the two PIK3CA^N345K^-specific TCRs, which showed much higher avidity to the mutant PIK3CA peptide. While these CD4 + T cell-derived TCRs showed limited tumor killing in a direct cytotoxicity assay in vitro, there is accumulating evidence that CD4 + *T* cells can execute tumor cytolysis by several other indirect mechanisms [20, 21]. Such mechanisms include the interaction of CD4 + *T* cells with stromal antigen presenting cells, which present tumor antigens from the TME, leading to activation and secretion of inflammatory and cytostatic cytokines such as IFN-γ and TNF-α. These cytokines can either have a direct cytotoxic effect on tumor cells and/or promote the activation and recruitment of tumor-reactive CD8 + *T* cells and iNOS-expressing monocytes and macrophages, which can further target and eliminate tumor cells. Therefore, CD4 + *T* cells can act as an essential orchestrator of the antitumor immunity in the TME, even in the absence or in addition to direct CD4 + *T* cell direct tumor cytotoxicity. Thus, tumor regressions have been observed in patients with advanced cancers following treatment with exclusively CD4 + *T* cells or HLA-class II restricted TCR. Previous clinical studies have shown that genetically engineered PBL with an HLA-class II receptor against MAGE-A3 mediated objective responses including two long-term complete responses [6] in patients with metastatic solid cancers. In addition, patient cases of durable clinical responses following TIL or TCR-engineered PBL ACT with exclusively or predominantly HLA-class II restricted targets have also been recently reported [1, 3, 10, 22]. One of TCRs described in this report was tested clinically, when patient 4367 received adoptive cell transfer treatment of autologous CD4 + enriched PBL, retrovirally transduced with the 4367_TCR-1 against PIK3CA^N345K^ [10]. At the first follow up (6 weeks post-ACT) the patient showed progression of disease and no further follow up occurred. Further evaluation of these PIK3CA-specific TCRs in patients with epithelial cancers is needed and to this goal, additional strategies can also be employed to enhance the potency of these TCRs in vivo. Such strategies can include gene modification of the *T* cells to sustain a favorable phenotype, such as induced overexpression of KLF2 and BACH2, and combination therapies, such as co-administration of immune-checkpoint blockade agents and neoantigen vaccination. These are active areas of research in our laboratory and their potential to augment the *T* cell efficacy is currently evaluated.

PIK3CA has been an immunologically elusive target and detection of naturally occurring *T* cells recognizing the mutant protein has been challenging. This is also evident in the number of patients with diverse cancer diagnosis and different PIK3CA mutations that we investigated by several approaches. Those approaches included TIL antigen-recognition screening and memory *T* cell (TIL and PBL) IVS with specific PIK3CA TMGs, long peptides and predicted minimal peptides. Despite a significant number of other neoantigens have been identified in the majority of this group of patients [1, 10, 15], often multiple within the same patient, only two of them were found to contain *T* cells against the mutant PIK3CA following our thorough investigation. It thus appears that PIK3CA lacks the features that set this molecule immunogenic to the degree that has been seen, by following similar investigational approaches, for other main cancer hotspot mutations, such as those in the TP53 and KRAS genes [4, 7, 16].

Despite this challenge, the current report provides evidence that PIK3CA-specific *T* cells can be identified and their TCR can be isolated from antigen-experienced TIL and PBL of patients with cancer. A clinical trial of off-the-shelf PIK3CA-specific TCR treatments for patients with epithelial cancers is required to determine the efficacy of immunologically targeting the mutant PIK3CA.

## Materials and methods

### Study participants

In this report, all patients had measurable metastatic cancer of epithelial origin and were 18 years of age or older. All patients were enrolled on a cell harvesting protocol prior to collection of lymphocytes or surgical resection (ClinicalTrials.gov: NCT00068003). Patient 4367 was enrolled into an ongoing early phase II pilot study designed to evaluate the safety and efficacy of ACT of autologous PBL genetically modified via retroviral transduction to express personalized neoantigen reactive TCRs after nonmyeloablative lymphodepleting chemotherapy in patients with metastatic epithelial cancer (ClinicalTrials.gov: NCT03412877).

These studies were approved by the Institutional Review Board (IRB) of the National Cancer Institute. Informed consent was obtained from all patients and all studies were conducted in accordance with the International Conference on Harmonization Good Clinical Practice and the applicable portions of the U.S. Code of Federal Regulations.

### TIL generation

TIL were generated as previously described [23]. Briefly, resected metastatic deposits were dissected in small fragments (1–2 mm), placed individually into wells of a 24-well plate in the presence of complete media (CM: RPMI 1640 medium (Gibco) supplemented with 10% human serum (Valley Biomedical), 25 mM HEPES (Gibco), 2 mM L-glutamine or Glutamax-I (Gibco)) containing high dose IL-2 (6000 IU/ml) (Peprotech) and antibiotics (penicillin G (100 units/ml), streptomycin (100 μg/ml) and gentamicin (50 μg/ml)) (Gibco). TIL were cultured at 37 °C with 5% CO2 until sufficient cell numbers (usually 4 confluent wells of a 24-well plate) were obtained for cryopreservation (Cryostor® CS10, Biolife Solutions).

### Whole exome sequencing (WES), RNA sequencing and determination of genomic variants

Genomic DNA (gDNA) and total RNA was purified from snap frozen fresh tumor and autologous apheresis samples, which was used as normal tissue controls. Whole-exome and RNA library construction, exon capture, and next-generation sequencing was performed as described previously [1, 15, 22]. Data processing, transcript alignment, variant calling and curation of mutations for screening was performed as has previously described [1, 15, 22].

### Minimal epitope prediction

HLA-class I predicted minimal epitopes with binding affinity ranking ≤ 2 were curated for testing by using the netMHCpan3-0 binding algorithm [24]. Specifically, 8-12AA long peptides were sorted based on their predicted binding affinity to a set of HLA-class I molecules from a group of patients enrolled in our protocols who also contained tumors with the PIK3CA^E545K^ mutation (Table S5). The selected seven minimal epitopes are listed in Fig. 3B and Table S5.

### Preparation of Antigen Presenting Cells (APCs) for TIL Screening

Immature dendritic cells were generated from peripheral blood derived monocytes as has been previously described [1]. Briefly, mononuclear cells from patient apheresis were incubated for 2 h in T175 flasks at 37 °C with 5% CO2 for adherence. Following extensive washing and an additional 1-h incubation, the adherent monocytes were washed twice and incubated for 72–96 h in dendritic cell (DC) media (RPMI 1640 medium supplemented with 5% human serum, 2 mM L-glutamine) supplemented with 800 IU/ml GM-CSF (Peprotech) and 200 U/ml IL-4 (Peprotech).

### *Generation of tandem minigene (TMG) constructs and *in vitro* transcribed (IVT) RNA*

Tandem minigenes were constructed as previously described [3]. Fourteen to seventeen minigenes were included in each TMG construct for this study. Plasmids encoding the minigenes were linearized with the restriction enzyme NotI (New England Biolabs) and each linearized plasmid was used as a template for in vitro transcription using the mMESSAGE mMACHINE® T7 Transcription Kit (Invitrogen), following the manufacturer’s instructions.

### RNA transfection and peptide pulsing of autologous APCs

Dendritic cells (APCs) were transfected with TMG RNA using the ECM830 square wave electroporator (Harvard Apparatus), as it has been previously described [1, 15, 22]. Briefly, IVT RNA (8–10 μg) was mixed with 100 μl of the APC suspension (0.5–3.5e6 cells/100 μl OPTI-MEM (Gibco)) just before electroporation is performed at optimized conditions for dendritic cells (150 V, 10 ms). Following electroporation, cells were immediately transferred into tubes containing DC media, supplemented with 800 IU/ml GM-CSF and 200 U/ml IL-4, at concentration 1e6 cells/ml and incubated overnight at 37 °C, 5% CO_2_.

All peptides were synthesized by Genscript or in the Surgery branch, reconstituted in DMSO in stock concentration 50 mg/ml or approximately 27 mg/ml, respectively and were used to pulse APCs either individually or as a mixture of peptides (peptide pools). For the 4367 TIL screen, the peptide pools consisted of 10–11 long peptides, each representing a mutated variant. The peptide pool of predicted short peptides for 4211 contained 7 minimal epitopes as shown in Fig. 3B. Autologous dendritic (APCs) incubated with 10 µg/ml (unless otherwise indicated) of each peptide for 2–4 h, at 37 °C, 5% CO_2_. Following pulsing, the APCs were centrifuged, resuspended in fresh CM, without cytokines and used immediately in co-culture assays.

### T cell activation measurement: IFN-γ ELISPOT and flow cytometry for activation marker 4-1BB and OX-40

*T* cell activation by putative antigens was assessed by IFN-γ ELISPOT assay and flow cytometry for the measurement of the surface proteins 4-1BB (CD137) (BD Biosciences) and/or OX-40 (CD134) (BD Biosciences), as previously described [1, 15, 22].

Briefly, APCs electroporated or pulsed with TMGs and peptides respectively, were co-cultured with *T* cells in CM media without cytokines for 18-24 h in indicated E:T ratios, or otherwise in a typical ratio 1:2. When the secretion of IFN-*γ* was to be measured, the co-culture was set in PVDF membrane 96-well plates coated with IFN-*γ* capture antibody D1K. After co-culture the cells were transferred into a new plate for flow cytometry antibody staining and the ELISPOT plate was washed and the spots developed by using sequentially, biotin-conjugated IFN-*γ* detention antibody, streptavidin-ALP, and BCIP/NTP as previously described [22].

For flow cytometry, following co-culture with APCs, the cells were stained with the appropriate antibody mix for 30 min in 4 °C and sequentially washed and resuspended in cold FACS buffer (PBS (Gibco) supplemented with 2% FBS and 2 mM EDTA (Quality Biological)). All data acquisition was performed in BD FACSCanto II or FACSymphony A3 flow cytometer (BD biosciences) and data analysis was performed using the FlowJo software (FlowJo, LLC). All data was gated on Lymphocytes (FSC-A vs SSC-A), single (FSC-H vs FSC-W and SSC-H vs SSC-W) and live (PI or DAPI negative) cells.

### TCR identification and construction

#### Single cell RT-PCR

The single cell RT-PCR protocol for the identification of neoantigen-reactive TCR pairs has been previously described [25]. Briefly, 4-1BB + and/or OX-40 + *T* cells were sorted into RT-PCR mix, following co-culture with neoantigen-pulsed APCs. Following a reverse transcription and first amplification reaction, a multiplex PCR with multiple Vα and Vβ region primers was performed, using the first RT-PCR products as templates. The final PCR products were sequenced by Sanger method with an internally nested *Cα* or *Cβ* region primer.

#### Single-cell next-generation sequencing

Single-cell TCR sequencing was performed using the Takara SMARTer® Human scTCR a/b Profiling Kit – 96 (Takara Bio) according to manufacturer instructions. Briefly, single cells from populations of interest were sorted into wells of a 96 well plate and subjected to cDNA synthesis and amplification followed by further amplification of the TCR-alpha and TCR-beta transcripts. Libraries were sequenced on an Illumina MiSeq® instrument using the MiSeq Reagent Kit v3 (600 cycle) (Illumina) to generate paired-end, 2 × 300 bp reads. The MiXCR software package was used to generate read extraction and clonality counts (https://milaboratory.com/software/mixcr/).

#### Full length TCR construction

Full length TCR were constructed by fusing the CDR3 region of a putative reactive TCR (provided by the RT-PCR, TCR sequencing analysis) with the partly missing conserved V and J region sequences, obtained from the online database IMGT (http://www.imgt.org), as previously described [22]. Alternatively, the full length TCR could be obtained by the sequencing analysis of the TCR-focused NGS sequencing. The resulting human V-(D)-J region from either method was directionally cloned into a MSGV1 retroviral vector that contains a modified mouse TRAC and TRBC region. Within the vector, the alpha and beta chain are separated by a furin SGSG P2A linker.

#### TCR Transduction of Peripheral Blood T Cells

Transduction of autologous or allogeneic peripheral blood *T* cells was conducted as has previously described [26]. Transduced *T* cells were evaluated for transduction efficiency at day + 7 post-activation by flow cytometry using anti-CD3 and anti-murine TCR β-chain (mTCR) (BD Biosciences) staining.

#### TCR specificity

All TCRs verified for specificity to the indicated PIK3CA antigen using HPLC (≥ 95%) mutant and wild type peptides (Genscript) either at 10 μg/ml or at a series of concentrations (titration assay: 10, 1, 0.1, 0.01 and 0.001 μg/ml).

### In vitro stimulation (IVS) of peripheral memory T cells

IVS was performed with the peripheral memory *T* cells as previously described [17]. Briefly, memory *T* cells were sorted from patient apheresis by FACS based on the expression of the surface markers CD62L and CD45RO [CD62L + CD45RO- (Tnaive), CD62L + CD45RO + (Tcm), CD62L-CD45RO + (Tem) and CD62L-CD45RO- (Temra)]. A sorting shoulder gate, excluding the Tnaive was drawn and memory CD4 + and CD8 + *T* cells were sorted separately. Then, they were subjected to stimulation by APCs electroporated with PIK3CA-specific TMG or pulsed with the relative mutant PIK3CA long peptide (25AA) or a pool of the predicted minimal epitopes. At the end of IVS, the *T* cell cultures were screened against each of the individual stimulating agents (TMG, long peptide or minimal epitopes pool) for detection of *T* cell reactivity against the mutated PIK3CA. Reactive cells were sorted by flow cytometry and were either subjected to expansion by REP (Pt. 4367) or submitted to a second round of IVS, followed by a second screen for PIK3CA-specific reactivity (Pt. 4211). Finally, reactive *T* cells were sorted for single cell sequencing and the identification of the reactive TCR pair.

All peptides used during IVS stimulation and screen were HPLC (> 95%) purified (Genscript). However, different batches of synthesized peptides were used between stimulation and screen to avoid a false positive result to any potential peptide synthesis byproduct.

### Identification of HLA-restriction by transfection of COS-7 cell lines with individual HLA molecules

The identification of the HLA-class II restriction for each of the TCRs were performed as previously described [22]. Briefly, each of the patient’s HLA molecules was introduced transiently and separately into COS-7 cells (ATCC), pulsed with the identified PIK3CA neoantigen and co-cultured with TCR-transduced *T* cells for activation assessment. In this transient introduction of HLA on COS7, the HLA-surface expression is not assessed by flow cytometry. Initially, COS7 cells were plated on a 96 well flat bottom plate and the next day they were transfected with HLA-class II allele plasmids (150 ng each), matched to the HLA alleles expressed by the patients 4211 and 4367 or no plasmid (mock) for negative control. The following day, the media was replaced with fresh, and the cells were directly proceeded to PIK3CA peptide (or DMSO as vehicle) pulsing for 4 h. Following the incubation, the wells were washed 3 × with media and were either proceeded to the co-culture with *T* cells or transferred to an ELISPOT plate for IFN-*γ* secretion assay. If transferred, the pulsed COS7 were detached by 30 incubation with cold PBS (w/o Ca^2+^ and Mg^2+^) in 4 °C for 30 min, followed by repeated pipetting and transfer into an IFN-*γ* antibody-pre-coated ELISPOT PVDF-membrane 96-well plate. Gene engineered PBL with PIK3CA- specific TCR were added to E:T ratio 1:1 in media without cytokines and placed at 37 °C, 5% CO_2_ for 18–20 h. Next day, activation of *T* cells was accessed by flow cytometry (upregulation of 4-1BB surface marker) or IFN-*γ* ELISPOT assay, as described above for *T* cell activation measurement.

### Flow cytometry antibodies

The following anti-human flow cytometry antibodies were used in our study: CD3-APC-H7 (clone: SK7), CD137(4-1BB)-APC (clone: 4B4-1), CD134(OX-40)-PE (clone ACT35), CD4-FITC (clone: RPA-T4), CD4-PE (clone: RPA-T4), CD8-PE-Cy^TM^7 (clone: RPA-T8), CD62L-PE (clone: DREG-56), CD45RO-APC (clone: UCHL1), HLA-DR-PE (clone: L243) and HLA-DP-PE (clone: B7/21) (BD Biosciences) and CD4-BV605 (clone: ΟΚΤ4) (Biolegend). Also, the following anti-mouse flow cytometry antibodies were used: TCRβ chain-FITC (clone: H57-597) (BD Biosciences).

### Tumor cell lines

COS-7, and HEK-293 cells (ATCC) were cultured in DMEM (Gibco), supplemented with 10% FBS (Gibco) without any antibiotics.

HCT-15 (CCL-225) cell line (ATCC) was cultured in RPMI 1640 media. HTB-22 (MCF-7) and HTB-114 tumor cell lines (ATCC) were cultured in Eagle’s Minimum Essential Medium (EMEM) (Gibco). All the tumor cell media were supplemented with 10% FBS, 1% sodium pyruvate, 1% Glutamax, 1% minimum essential medium (MEM) nonessential amino acids, 55 mmol/L 2-mercaptoethanol, and 1% Pen/Strep (Gibco). All the cell lines were used between passages 5–15 and mycoplasma testing was performed routinely.

HTB-114, MCF-7 and CCL-225 cells lines were also modified to express HLA-restriction molecule of interest by retroviral gene transfer. Specifically, the full length of the HLA molecules DRB1*04:01, DPB1*04:01 or A*03 (AA sequences obtained from IPD-IMGT/HLA), synthesized and cloned into pMSGV1 vector by Genscript, were packed into retroviral particles using the HEK-293GP packaging cell line with a RD114 envelope, similar to the process followed for the TCR transduction described above. Similarly, for the cell lines HTB-114 and CCL-225 the full length of mutated (p.N345K) or wild type PIK3CA gene (GFP-tagged) (NM_006218.4) was retrovirally transferred. Each gene transfer was performed in two consecutive days of retroviral infection of the target cells. HLA-DR + or DP + transduced cells with overexpression of HLA-DR + and -DP molecules (over mock transduced control) and GFP positive signal were sorted and expanded before testing. Expression of the appropriate HLA and GFP were routinely verified by flow cytometry analysis and/or functional assays.

### Reporter cell lines generation

Red fluorescent protein expressing tumor cell lines for in vitro cytotoxicity assays were generated by using the NucLight Red lentivirus system (Essen BioScience). Briefly, engineered tumor cell lines MCF-7, CCL-225, HTB-114 cells were transduced with Nuclight Red Lentivirus (EF1a, Puro) at multiplicity of infection (MOI) of 5 with 8 μg/ml polybrene. After 24 h at 37 °C, cells were washed with fresh media and cultured for an additional 7 days in the presence of 2 μg/ml puromycin (Gibco) as selection agent.

### Determination of tumor cell killing by live cell imaging

Incucyte (Sartorius, Essen Bioscience) and CellCyte X (Cytena, BICO) live-cell imaging systems were used for real-time imaging of RFP expressing tumor cells. 5000 tumor cells were seeded in 100 μl of complete culture medium in the 96-well plate and rested overnight at 37 °C and 5% CO2. Next day, TCR-transduced PBL were added (100 μl) on top of tumor cells at E:T ratio 10:1. Red cell count per field of view (10 × magnification, 4 images/well) was measured over 4 h intervals, for 96–100 h. Each test condition was performed in 6 replicates. Images were analyzed using IncuCyte 2021B software and data were generated using the GraphPad Prism statistical software. The data were reported as mean ± S.D. and each experiment were performed at least twice.

### Statistical analysis

Any descriptive statistics, such as median values, their standard deviation and EC50 values, were all performed using GraphPad Prism 10.1.1.

For the tumor killing assays, Mann–Whitney U test was performed following the calculation of the slopes between each time point of growth curves between the test (PIK3CA-reactive) TCRs and control (Irrelevant) TCR.

## Supplementary Information

Below is the link to the electronic supplementary material.

Supplementary file1 (DOCX 585 KB)

## Data Availability

All data relevant to the study are included in the article.
