# Supplementary material for: Identification of HLA class II-restricted T cell receptors against shared PIK3CA mutations in patients with epithelial cancers
Source: Cancer Immunol Immunother. 2025 Dec 18;75(1):5. doi: 10.1007/s00262-025-04156-3 (PMC12715084; doi:10.1007/s00262-025-04156-3)
Supplement: Supplementary file 1 — Supplementary file1 (DOCX 585 KB) [file 262_2025_4156_MOESM1_ESM.docx]

**Fig. S1: Identification of PIK3CA-N345K-specific TCRs in TIL and peripheral blood from a patient with colon cancer.**

1. 4-1BB (CD137) upregulation by CD3+/mTCR+ PBL from healthy donor-2, retrovirally transduced with 4367_TCR-1 in response to COS7 cells transfected with each of the 4367 class II HLA molecules and pulsed with the PIK3CA^N345K^ peptide or vehicle (DMSO).
2. 4-1BB+ (CD137) upregulation by 4367 memory CD4+ PBL following one round of IVS with PIK3CA^N345K^, and overnight co-culture with autologous DCs electroporated by either PIK3CA^N345K^ TMG or vehicle (control) (4-1BB+: 1.4% and 0.8%, respectively). Gated on Lymphocytes/Singlets/Live/CD3+/CD4+.
3. 4-1BB (CD137) upregulation of CD3+/mTCR+ PBL from healthy donor-2, retrovirally transduced with 4367_TCR-2 in response to titrated concentration of mutant or wild type long PIK3CA^N345K^ peptide pulsed on autologous DCs.

**Fig. S2: Generation and characterization of HLA and gene engineered HTB-114 tumor cell lines as a target of 4367 pN345K TCRs**.

Flow cytometric analysis for the expression of HLA-DPA1*01:03/DPB1*04:01 (X-axis) and expression of wild type or mutant PIK3CA (p.N345K-GFP, Y axis) in HTB-114 tumor cell lines. Mock indicates control (empty vector transduced).

**Fig. S3: Identification of PIK3CA-E545K-specific TCR in the peripheral blood from a patient with rectal cancer**

1. 4-1BB (CD137) upregulation (of CD3+/mTCR+) of PBL from healthy donor-2, retrovirally transduced with 4211_TCR-1 in, in response to titrated concentration of mutant or wild type PIK3CA^E545K_epit-3^ peptide pulsed on autologous DCs.
2. IFN-γ secretion (ELISPOT assay) of PBL from healthy donor-2 either mock-transduced or with 4211_TCR-1 following overnight co-culture with modified (as indicated) MCF-7 cell line.

**Fig. S4: Generation of HLA engineered MCF-7 and CCL-225 tumor cell lines as a target of 4211 TCR.**

(A) Flow cytometric analysis for the expression of empty vector mock (left panel) and HLA-DRB1*04:01 (right panel) in MCF-7 tumor cell line naturally expressing mutant PIK3CA^E545K^.

(B) Flow cytometric analysis for the expression of empty vector mock (left panel) and an irrelevant HLA-A*03 (right panel) in MCF-7 tumor cell line naturally expressing mutant PIK3CA^E545K^.

(C) Flow cytometric analysis for the expression of empty vector mock (left panel) and HLA-DRB1*04:01 (right panel) in CCL-225 tumor cell line naturally expressing mutant PIK3CA^E545K^.


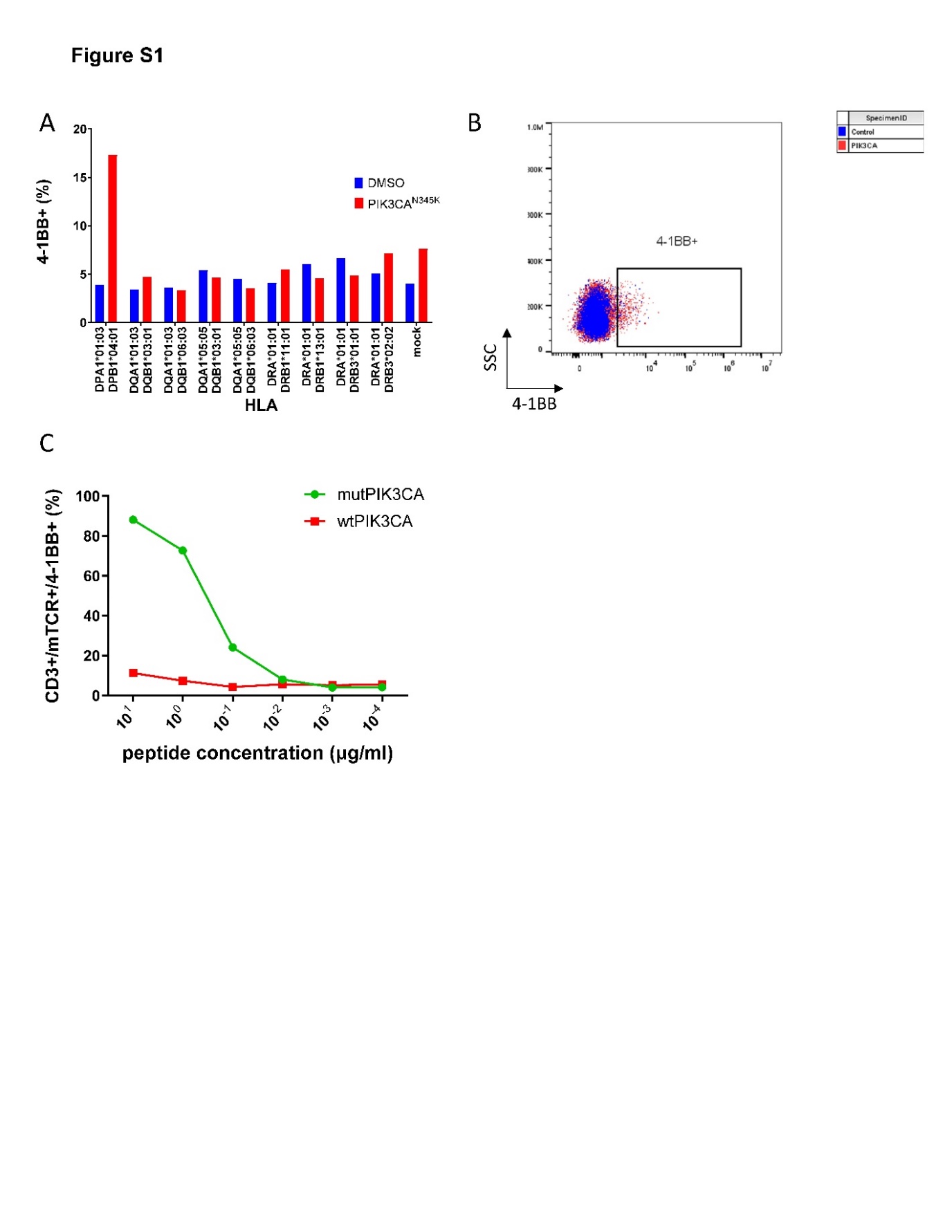

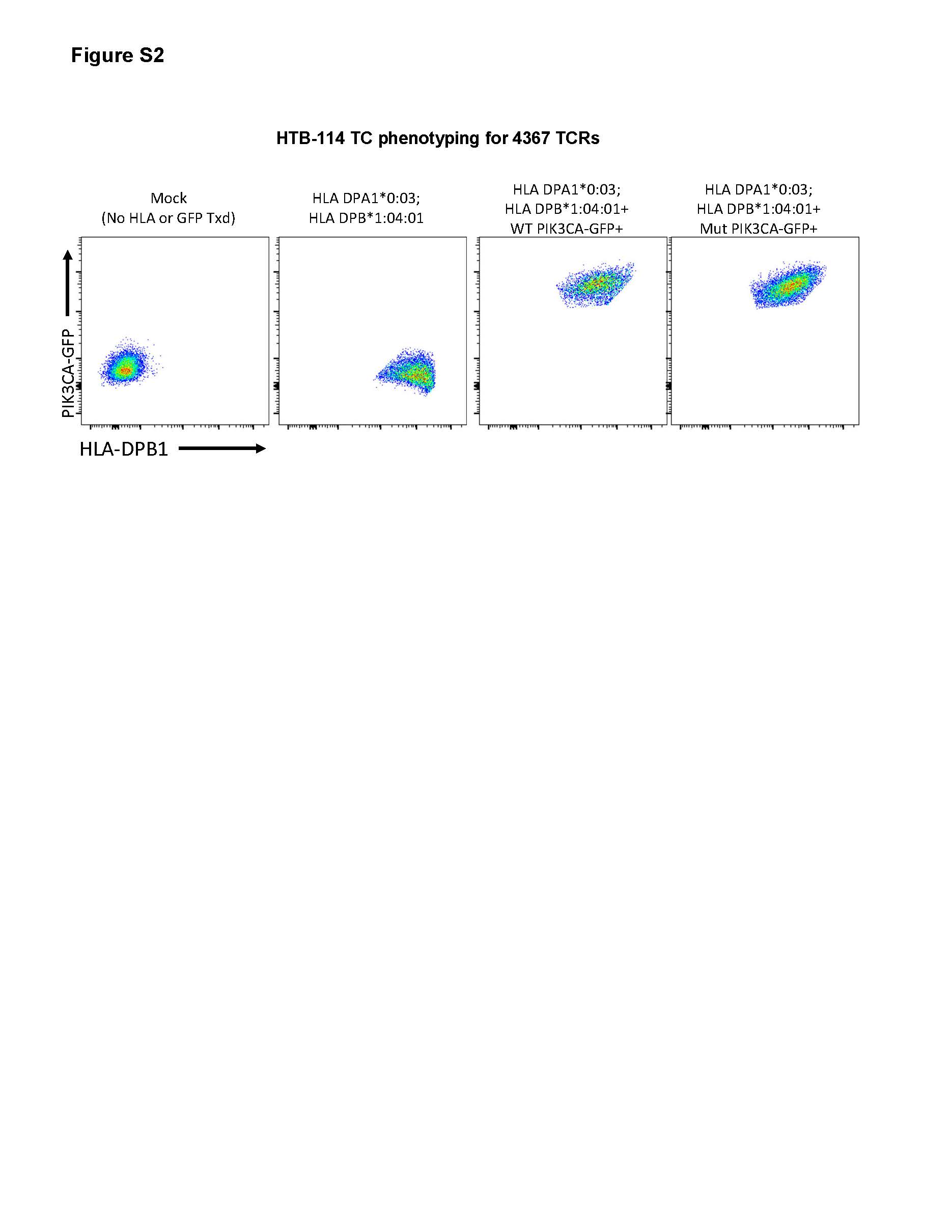
 **
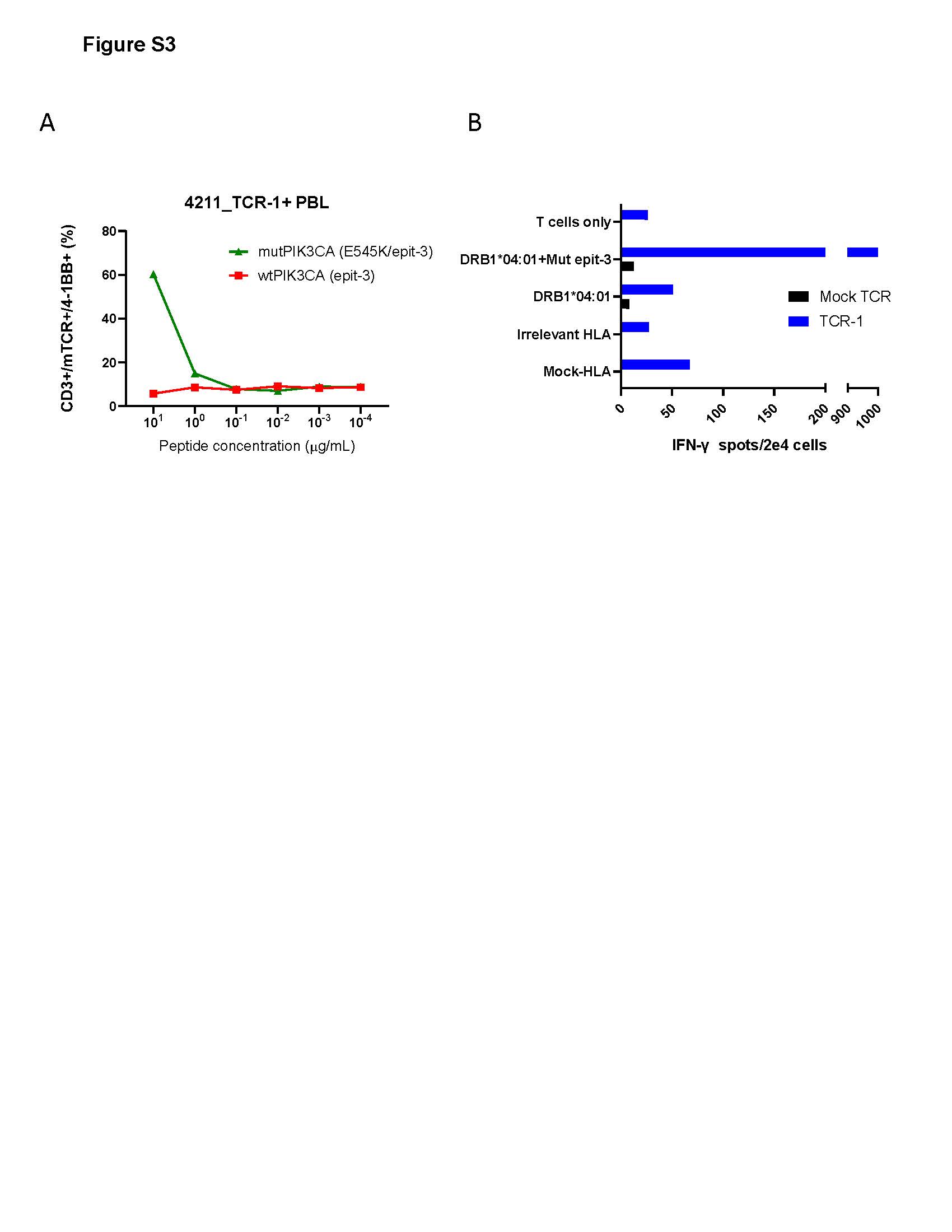

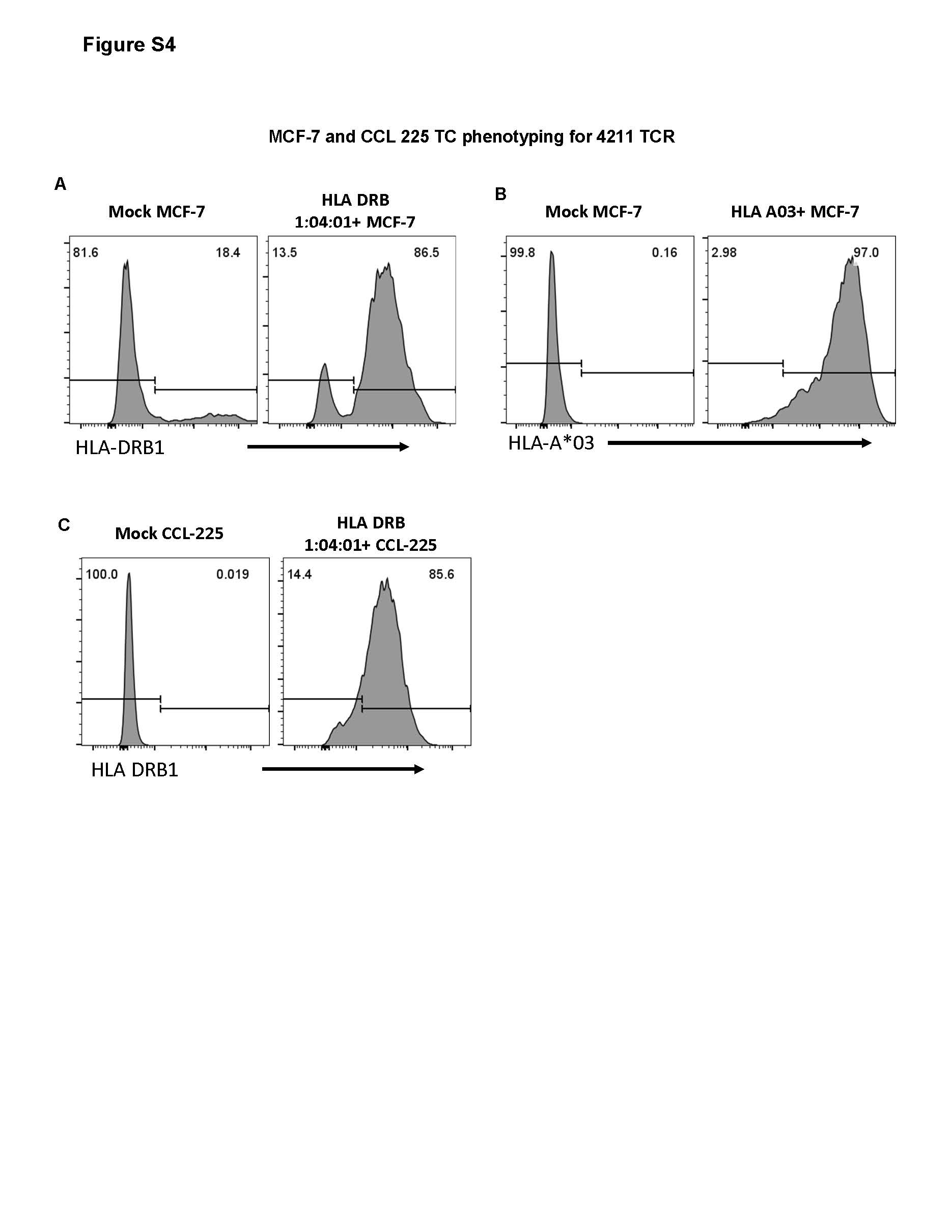
**
